# Astrocyte-Derived Tissue Transglutaminase Interacts with Fibronectin: A Role in Astrocyte Adhesion and Migration?

**DOI:** 10.1371/journal.pone.0025037

**Published:** 2011-09-16

**Authors:** Miriam E. van Strien, John J. P. Brevé, Silvina Fratantoni, Marco W. J. Schreurs, John G. J. M. Bol, Cornelis A. M. Jongenelen, Benjamin Drukarch, Anne-Marie van Dam

**Affiliations:** 1 Department of Anatomy and Neurosciences, VU University Medical Center, Neuroscience Campus Amsterdam, Amsterdam, The Netherlands; 2 Department of Pathology, VU University Medical Center, Neuroscience Campus Amsterdam, Amsterdam, The Netherlands; University of North Dakota, United States of America

## Abstract

An important neuropathological feature of neuroinflammatory processes that occur during e.g. Multiple Sclerosis (MS) is the formation of an astroglial scar. Astroglial scar formation is facilitated by the interaction between astrocytes and extracellular matrix proteins (ECM) such as fibronectin. Since there is evidence indicating that glial scars strongly inhibit both axon growth and (re)myelination in brain lesions, it is important to understand the factors that contribute to the interaction between astrocytes and ECM proteins. Tissue Transglutaminase (TG2) is a multifunctional enzyme with an ubiquitous tissue distribution, being clearly present within the brain. It has been shown that inflammatory cytokines can enhance TG2 activity. In addition, TG2 can mediate cell adhesion and migration and it binds fibronectin with high affinity. We therefore hypothesized that TG2 is involved in astrocyte-fibronectin interactions. Our studies using primary rat astrocytes show that intracellular and cell surface expression and activity of TG2 is increased after treatment with pro-inflammatory cytokines. Astrocyte-derived TG2 interacts with fibronectin and is involved in astrocyte adhesion onto and migration across fibronectin. TG2 is involved in stimulating focal adhesion formation which is necessary for the interaction of astrocytes with ECM proteins. We conclude that astrocyte-derived TG2 contributes to the interaction between astrocytes and fibronectin. It might thereby regulate ECM remodeling and possibly glial scarring.

## Introduction

Astrocytes within the brain are considered to be important for maintaining an environment in which neurons, other glial cell types and the brain endothelium function and interact properly [1]. Injury to the central nervous system (CNS) often results in a characteristic astroglial response, i.e. the astrocytes become activated, migrate, and form a dense network of hypertrophic cells, the astroglial scar [2]–[4]. Additional cell types including macrophages, microglia, oligodendrocytes, and meningeal fibroblasts contribute to the glial scar [5], but astrocytes predominate and are the focus of the present study. The astroglial scar consists of a fine meshwork of astrocyte processes strongly interwoven and bound together by tight and gap junctions, surrounded by extracellular matrix (ECM) [5]–[7].

In situations of chronic neuroinflammation, e.g. Multiple Sclerosis (MS), when inflammatory cytokines are produced and released within the CNS [8], sustained and excessive deposition of ECM proteins such as fibronection and activation of astroglial cells can create an environment in which an astroglial scar is formed. Moreover, the cytokine interleukin-1β (IL-1β) has been shown to promote the reactive astrocytic phenotype and adhesion of astrocytes onto fibronectin (Fn) or laminin [9]. The astroglial scar acts as a physical or biochemical barrier that impedes tissue repair [5]. For instance, a reduction in migration and differentiation of oligodendrocyte precursor cells (OPCs) has been described [10]–[12], as well as attenuated myelination of axons by oligodendrocytes [5].

Thusfar, studies on astrogliosis and down-stream mechanisms involved in the interaction between astrocytes and ECM molecules focus on relatively acute (hours) effects. However, patients suffering from neuroinflammation and/or brain injury experience long-term consequences of their disease i.e. impaired regeneration. In that respect, we are interested in the role of tissue Transglutaminase (tTG or TG2) in the interaction of astrocytes with ECM molecules, e.g. Fn after (relatively) long-term cytokine treatment. It has been shown that upon treatment of different cell types with cytokines, TG2 expression and activity was elevated for a longer period of time, i.e. up to 7 days [13], [14] which may be more reflecting the pathological situation in humans.

TG2 is an ubiquitous member of a family of Transglutaminase enzymes. Its functional role remains to be fully established, but TG2 is well known for its ability to posttranslationally modify proteins in a calcium-dependent manner. TG2 can cross-link proteins, amidate or deamidate proteins, it can bind and hydrolyse GTP to mediate cell signalling, and it has isopeptidase activity [15]. TG2 is mainly expressed intracellularly, but it can also be found extracellularly in the extracellular matrix [15]. In addition, it has been shown that TG2 is present on the surface of monocytes [16], monocyte-derived dendritic cells and macrophages [17], endothelial cells [18] and fibroblasts [19]. Since TG2 is present on the cell surface of these cell-types and has a Fn binding site located in the N-terminal domain, a prominent role for TG2 in mediating cell adhesion has been put forward [16], [19]. Besides the interaction of TG2 with Fn to facilitate cell adhesion, it is involved in numerous other adhesion-dependent phenomena including cell migration, extracellular matrix assembly [20], and cell signalling [21]. Cytoskeletal reorganization and focal adhesion dissolution [22], [23] are required to mediate those processes.

Based on these considerations, in our quest to unravel the mechanism(s) that contribute to relatively long-term astroglia activation and astrocyte-ECM interactions that could ultimately contribute to astroglial scar formation, we hypothesize that the enzyme TG2 is an important player. To investigate this issue, we used primary cultured rat astrocytes, and observed cytokine-induced enhanced intracellular and surface expression of active TG2. Furthermore, TG2-mediated cross-linking activity resulted in enhanced astrocyte adhesion onto and migration across Fn, most likely mediated via effects on focal adhesion-related vinculin.

## Materials and Methods

### Primary rat astrocyte culture and cytokine treatment

Primary cultures of astrocytes were prepared from newborn (2-day old) Dark Agouti rats (Harlan CPB, Zeist, The Netherlands) as described [24]. In short, cerebral cortices were cleared from adhering meninges and blood vessels and dissociated using 0.25% trypsin (Sigma-Aldrich, St. Louis, MO) in phosphate-buffered saline (PBS). Cells were plated in poly-L-lysine (15 µg/ml (0,4 µg/cm^2^/ml); Sigma-Aldrich) coated T75 culture flasks (Nunc, Hamstrop, Denmark) and incubated at 37°C in humidified air containing 5% CO2. The culture medium consisted of Dulbecco's modified Eagle's medium (DMEM)-F10 (Gibco, Life Technologies, Breda, The Netherlands), supplemented with 10% v/v heat-inactivated fetal calf serum (FCS) (Gibco), 2 mM L-glutamine (Sigma-Aldrich), 50 Units/ml penicillin (Sigma-Aldrich) and 50 µg/ml streptomycin (Gibco). The medium was changed 1 day after seeding. After 8 days of culture, pure astrocytes were obtained by shaking the flasks at 37°C on a rotary platform (Heidolph Unimax 2010) at 240 rpm for 16 h to remove microglia and oligodendrocyte progenitors. Astrocytes can be further purified by treatment (overnight (o/n), 37°C) treatment with 5 mM leucine methyl ester (LME, Sigma-Aldrich) in serum free medium. Fresh medium was added to the flasks and cells were ready for use in further experiments. Primary astrocytes were used for a maximum of 5 passages.

For experiments with primary rat astrocytes, cells were plated onto wells that were coated with 2 µg/mm^2^ Fn (Sigma-Aldrich) for 1 h at 37°C. Astrocytes were cultured in medium alone (control) or the presence of rat recombinant (rr) TNFα (50 ng/ml; Pharmingen, BD Biosciences, Breda, The Netherlands), rr IFNγ (50 ng/ml; R&D systems, Abingdom, United Kingdom), rr IL-1β, (50 ng/ml; gift from Glaxo IMB, Geneva, Switzerland) or combinations of the various cytokines (50 ng/ml each) for 48 h.

To determine the role of TG2 in adhesion to and migration over Fn, astrocytes were co-incubated with 0.5 mM KCC009, a specific irreversible inhibitor of TG2 activity [25] (gift from Alvine Pharmaceuticals, San Carlos, CA, USA), diluted in 0.2% DMSO or with 0.2% DMSO only (vehicle).

### Ethics statement

All experiments carried out in this study were strictly performed in a manner to minimize suffering of laboratory rats. The protocol was approved by the Animal Experiment Committee of the VU University Medical Center, Amsterdam, The Netherlands (approval ID: ANW 06-10).

### TG2 immunocytochemistry

Untreated primary astrocytes were cultured in Fn coated 8-well chamber slides (Nunc) at 37°C. After 24 h, cells were fixed with 4% paraformaldehyde in 0.1 M phosphate buffer (pH 7.6) for 15 min and subsequently rinsed with PBS. The cells were incubated with mouse anti-TG2 (Ab3, 10 µg/ml; Labvision) in PBS/2% bovine serum albumin (Sigma-Aldrich) for 4 h at 4°C. After washes in PBS, the cells were incubated with biotinylated donkey anti-mouse IgG's (1∶500; Jackson laboratories) for 1 h at room temperature (RT) followed by an hour incubation with Alexa Fluor 488-coupled streptavidin (1∶400; Invitrogen) at RT. Cells were washed with PBS and slides were embedded in vectashield (Vector Laboratories Inc., Burlingame, CA, USA). Pictures were taken using a Colorview II digital camera (Olympus, Soft Imaging System, Munster, Germany) and Cell*F software (Olympus Soft Imaging Solutions GmbH).

### Semi-quantitative RT- PCR

For semi-quantitative RT-PCR, 1×10^6^ astrocytes were homogenized in Trizol reagent (Invitrogen, Carlsbad, USA) and total RNA was isolated as described by the manufacturer. RNA concentration and purity was determined by measuring the absorbance at 260 nm and 280 nm in a microtiter plate reader (Spectramax 250, Molecular Devices). One µg of RNA was reverse transcribed into cDNA using the Reverse Transcription System (Promega, Madison, WI, USA) with oligo-dT primers and AMV enzyme, according to the manufacturer's instructions. The PCR reaction was carried out at 42°C for 30 min, followed by deactivation of the enzyme at 95°C for 5 min and 4°C for 5 min. For the PCR reaction, the SYBR Green PCR Core reagents kit (Applied Biosystems, Foster City, CA, USA) was used. Intron-spanning primers were designed using Primer Express Software (Applied Biosystems) and purchased from Eurogentec (Seraing, Belgium). Amplification of cDNA was performed in MicroAmp Optical 96-well Reaction Plates (Applied Biosystems) on an ABI PRISM 7700 Sequence Detection System (Applied Biosystems). The reaction mixture (20 µl) was composed of 1× SYBR Green buffer, 3 mM MgCl_2_, 875 µM dNTP mix, 0.3 U AmpliTaq gold, 0.12 U Amperase UNG, 15 pmol of each primer (Table 1), 12.5 ng cDNA and nuclease free H_2_O. The reaction conditions were an initial 2 min at 50°C, followed by 10 min at 95°C and 40 cycles of 15 sec at 95°C and 1 min at 59°C. The mRNA expression levels were quantified relatively to the level of the housekeeping gene glyceraldehyde-3-phosphate-dehydrogenase (GAPDH) using the following calculation:
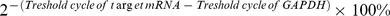



**Table 1:** Oligonucleotide primers used for amplification of cDNAs^1^.

rat cDNA	Sequence forward 5′ to 3′	Sequence reverse 5′ to 3′
GAPDH	TCAAGGGCATCCTGGGCTAC	CGTCAAGGTGGAGGAGTGG
TG1	ACCAGCAGTGGCATCTTC	AATGAAAGGTGTGTCATACTTC
TG2	AGAGGAGCGGCAGGAGTATG	AGGATCCCATCTTCAAACTGC
TG3	GAAGTCAAGGTGTGTTCC	AGATGAAGATCATGTCGAA
TG6	TCAATCCCTGGTGCCCAGAG	ACGCCTCGGAAGATGATGCC
FXIIIa	TCACTGCACGCATCAACGAGAC	TGCCTCGGACCTTGATGGTGAC

1 Primers were designed using the Primer Express program (Applied Biosystems) and purchased from Eurogentec (Seraing, Belgium).

### Western blotting

Astrocytes were homogenized in ice-cold lysis buffer containing 10 mM Tris/HCl pH 7.5, 150 mM NaCl, 1 mM EDTA, 1 mM DTT, 100 µM PMSF, 10 µg/ml leupeptin, 10 µg/ml pepstatin and 10 µg/ml aprotinin (all from Sigma-Aldrich). Homogenates were cleared by centrifugation at 14,000 rpm for 30 min at 4°C, and protein concentrations of supernatants were determined by the BCA method (Pierce Biotechnology, Etten-Leur, The Netherlands). Of each sample, 10 µg of protein was subjected to 10% SDS-polyacrylamide gel electrophoresis (SDS-PAGE) and transferred to a polyvinylidene difluoride (PVDF) membrane (Invitrogen, Carlsbad, CA, USA). Membranes were incubated with primary mouse anti-TG2 (Ab3, 1∶1,000; Labvision, Fremont, CA, USA), mouse anti-vinculin (1∶400; Abcam, Cambridge, UK), or mouse anti-β-actin (1∶2,000; Abcam). For subsequent antigen detection, blots were incubated for 2 h with corresponding goat anti-mouse Immunoglobulins/HRP (1∶1000; Dako, Glostrup, Denmark). Bands were visualized using the enhanced chemiluminescence (ECL) detection system SuperSignal West Dura (Pierce Biotechnology) and a Chemidoc image capture system (Bio-Rad, Veenendaal, The Netherlands). Signal intensity of the bands was semi-quantified using Quantity One software (Bio-Rad).

### TG2 ELISA

TG2 protein levels were measured in cell lysates using a sandwich enzyme-linked immunosorbent assay (ELISA) specific for TG2 as described previously [26]. Briefly, astrocytes were homogenized in ice-cold lysis buffer as described for western blot. Homogenates were cleared by centrifugation (14,000 rpm for 30 min at 4°C) and protein concentrations of supernatants were determined by the BCA method (Pierce Biotechnology). Of each sample, 10 µg of protein was loaded into the assay. An immunoaffinity-purified polyclonal goat anti-TG2 antibody (1∶1,000; Upstate, Millipore, USA) was used as coating antibody and a monoclonal mouse anti-TG2 antibody (Ab2, 1∶1,000; Labvision) was used as detecting antibody. Recombinant human TG2 (Zedira Biotec GmbH, Darmstadt, Germany) was used as a standard.

### TG activity assay

To measure TG activity, astrocytes were treated with cytokines at 37°C for 48 h. Subsequently, cells were homogenized in ice-cold lysis buffer as described for western blot. Homogenates were centrifuged for 30 min at 14,000 rpm at 4°C, and protein concentrations of supernatants were determined by the BCA method (Pierce Biotechnology). Activity was measured by using the TG Covtest TCMA (Transglutaminase Colorimetric Microassay; Covalab, Villeurbanne, France) following manufacturer's protocol [27]. In short, immobilized CBZ-Gln-Gly was coated onto the wells as the first TG substrate. Subsequently, 10 µg of protein from each sample was added/well, followed by addition of biotinylated cadaverine as a second TG substrate. After 30 min incubation at 37°C, plates were washed with Tween-20 buffered saline (TTBS) and 100 µl streptavidin-labelled peroxidase (HRP) diluted in TTBS was added to the wells for 15 min. After washing, peroxidase activity was revealed using 100 µl of 0.01% H_2_O_2_ as HRP substrate and (0.1 mg/ml) tetramethyl benzidine as electron acceptor (chromogen). The reaction was stopped by the addition of 50 µl of 2.5 N H_2_SO_4_. TG activity was detected by absorbance measurement of streptavidin-labelled peroxidase activity in each well on a microplate reader (SpectraMax 250, Molecular Devices) at 450 nm. Guinea pig TG2 (T5398, Sigma-Aldrich) was used as a standard. One unit of guinea pig TG2 will catalyze the formation of 1.0 µmole of hydroxamate per min from Nα-Z-Gln-Gly and hydroxylamine at pH 6.0 at 37°C.

### Detection of immunofluorescent TG2 on the cell surface

For immunofluorescent surface labeling of TG2, untreated astrocytes were washed with TBS and incubated on ice with a mouse monoclonal anti-TG2 antibody (Ab1, 1∶1,000; Labvision) for 2 h. Cells were washed with TBS, followed by incubation for 1 h on ice with goat anti mouse coupled to Alexa Fluor-488 (1∶400; Invitrogen). Subsequently, cells were washed with TBS and fixed for 10 min with 4% paraformaldehyde (PFA). After washing with H_2_O, sections were embedded in Vectashield mounting medium (Vector Laboratories) and examined on a Leica confocal laser scanning microscope (Leica, Rijswijk, The Netherlands).

### Cell surface biotinylation and immunoprecipitation

To determine TG2 expression on the cell surface, cells were plated in Fn coated wells of a 6-well plate (2.5×10^5^ cells/well, Nunc), and allowed to adhere o/n. Cells were incubated with cytokines (50 ng/ml) for 48 h. Subsequently, cells were washed with ice-cold PBS. Cell surface proteins were biotinylated by incubating the astrocytes for 20 min with 0.2 mg/ml Sulfo-NHS-LC-biotine (Pierce Biotechnology) in PBS at 4°C. The reaction was stopped by addition of 100 mM mM Tris-HCl, pH 7.5. Surface-biotinylated cells were washed with in PBS at 4°C and lysed in ice-cold lysis buffer as described for western blot. Cell lysates were cleared by centrifugation (14,000 rpm for 30 min at 4°C). Then, 100 µg of total cell protein was taken for each immunoprecipitation. Cell lysates were incubated with 100 µl 50% Neutravidin agarose beads (Pierce Biotechnology, pre-washed with TBS/1% SDS) for 3 h at RT. Beads were washed with TBS/1% SDS and then with TBS. Beads were collected in 4× LDS sample buffer (Invitrogen) containing 50 mM DTT and boiled for 10 min. Samples were centrifuged for 5 min at 12,000 rpm at 4°C and levels of surface TG2 were detected using gel-electroforesis and western blot as described above.

### Flow cytometry

To study TG activity on the surface of astrocytes after treatment with various cytokines and the effect of inhibition of TG2 activity, the incorporation of a biotinylated primary amine substrate 5-(biotinamido) pentylamine (BAP) of TG was measured using flow cytometry (FACS). Detection of BAP by FACS analysis reflects TG activity on the cell surface of astrocytes. Therefore, astrocytes were plated onto Fn coated wells and cultured in the presence of TNFα, IL-1β or the combination of both for 48 h. After incubation, cells were washed with PBS containing 0.1% glucose and detached with 10 mM EDTA in PBS/0.1% glucose. Subsequently, cells were counted and 1.5×10^5^ cells were resuspended in 1 ml AC-buffer containing PBS/0.1% glucose containing 1.2 mM CaCl_2_ and 0.5 mM MgCl_2_. Then, cells were pre-incubated with 0.5 mM KCC009 or vehicle in AC-buffer at 37°C for 15 min. Finally, BAP (Pierce Biotechnology) was added to the cells in a final concentration of 0.2 mM and cells were co-incubated for 1 h at 37°C with vehicle or KCC009. After incubation, cells were placed on ice, washed with AC-buffer containing 0.1% BSA and subsequently stained with streptavidin-Alexa Fluor-488 (1∶300; Invitrogen) at 4°C for 30 min. Cells were washed with AC-buffer containing 0.1% BSA and resuspended in AC-buffer/0.1% BSA containing 1 µg/ml propidium iodide (PI, Sigma) to stain apoptotic cells. Then, flow cytometry was performed on a FACScan (BD Biosciences) and data were analyzed using The Windows Multiple Document Interface (WinMDI) software (freeware designed by J. Trotter to analyze flow cytometric listmode data files).

Analysis gates were set on propidium iodide (PI) negative cells. Three independent experiments were performed and in each experiment, BAP+vehicle-treated control cells were set at 100%.

### BAP incorporation assay

To study whether active TG2 is involved in the interaction between astrocytes and Fn, the incorporation of the biotinylated amine substrate BAP into Fn was measured [28]. In short, 48 h-cytokine treated rat astrocytes were detached with 10 mM EDTA, resuspended in fresh medium and plated onto Fn coated 96-well plates (50 µl/well corresponding to ∼3.5*10^4^ cells/well) in the presence of 0.5 mM KCC009 in 0.2% DMSO or 0.2% DMSO only (vehicle). BAP was added directly (0.1 mM; Pierce Biotechnology) and cells were incubated for 1 h at 37°C. Subsequently, cells were washed with PBS containing 3 mM EDTA and then incubated for 20 min with 0.1% sodiumdeoxycholate (Sigma) in PBS containing 3 mM EDTA. Supernatant and cells were removed and the plate was washed with 0.1 M Tris-HCl (pH 7.4). Then, the plate was incubated for 1 h at 37°C with streptavidin poly-HRP (1∶10,000 in Tris/HCl). After washing with Tris/HCl, peroxidase activity was revealed using 0.01% H_2_O_2_ and 0.1 mg/ml tetramethyl benzidine (chromogen). The reaction was stopped by the addition of 50 µl of 2.5 N H_2_SO_4_ and OD was measured on a microplate reader (SpectraMax 250, Molecular Devices) at 450 nm. Three independent experiments with duplicate measurements were performed for each treatment.

### Astrocyte adhesion onto Fn

Astrocyte adhesion was studied as described [29]. In short, 96-well plates were coated with Fn (Sigma-Aldrich) for 1 h at 37°C. Primary rat astrocytes that had been treated with cytokines for 48 h were detached with 2 mM EDTA and plated onto Fn-coated wells (5×10^4^ cells/well) in serum free medium. Cells were allowed to adhere for 3 h at 37°C in the presence of 0.5 mM KCC009 or vehicle. After 3 h, cells were washed with PBS and fixed with 4% PFA in 0.1 M phosphate buffer for 30 min at RT. Cells were stained with 100 µl crystal violet solution (0.5 gr/100 ml 70% EtOH) for 40 min. Cells were washed with PBS and crystal violet was extracted from the cells with 100 µl 30% acetic acid. The absorbance was measured on a microplate reader (SpectraMax 250, Molecular Devices) at 540 nm. Three independent experiments with duplicate measurements were performed for each treatment.

### Astrocyte viability measurements

The effect of KCC009 treatment on cell viability was determined by PI exclusion assay. Rat astrocytes were plated in a 96-well plate (20,000 cells/well) and allowed to adhere for 24 h in serum free medium. Medium was replaced with PBS containing 0.5 mM MgCl_2_, 1.2 mM CaCl_2_, 0.1% glucose, 40 µg/ml PI (Sigma) and 0.5 mM KCC009 or vehicle. During an incubation period of 24 h at 37°C, PI fluorescence was measured at 30 min intervals, using a Fluostar OPTIMA microplate reader with an excitation wavelength of 544 nm and an emission of 612 nm. Average slope/minute was measured per 30 minute interval and average slope/minute in 24 h was determined (F-average). A total of 160 µM digitonin was then added for 20 min to permeablize all cells and fluorescence measurements were performed to obtain a maximal fluorescent signal (Fmax). Percentage viability was calculated as: 100−(F-average/Fmax-blank)×100% [30].

### Astrocyte migration across Fn

Astrocyte migration was studied as described [29]. In short, permanox Chamberslides (4-wells, Nunc) were coated with 2 µg/cm^2^ Fn (Sigma-Aldrich) for 1 h at 37°C. Primary rat astrocytes were plated onto the Fn-coated wells (2×10^5^ cells/well) and allowed to adhere for 24 h. Then, cytokines were added to the cells. After 24 h, 0.5 mM KCC009 or vehicle was added to the cells in serum-free medium and a scratch wound was made in each well by using a sterile 10 µl pipet-tip (Corning). At 0 and 16 h after wound induction, cells were fixed with 4% PFA for 20 min. Cells were washed with PBS and subsequently stained with rhodamine-phalloidin (1∶300; Invitrogen) for 1 h at RT, washed with PBS and embedded in vectashield (Vector Laboratories). Pictures were taken using a Colorview II digital camera (Soft Imaging System, Gmbh, Germany). The diameter of the wound (where no cells were present) was measured using Cell*F software (Olympus Soft Imaging Solutions GmbH, Germany). Five random 20× fields per culture condition were captured, and mean wound diameter was assessed in each field and averaged over the five fields.

### Bromodeoxyuridine labeling assay

To determine the effect of KCC009 treatment on astrocyte proliferation, primary rat astrocytes were plated onto the Fn-coated wells (2×10^5^ cells/well) and allowed to adhere for 24 h. Then, cytokines were added to the cells. After 24 h, 0.5 mM KCC009 or vehicle was added to the cells in serum-free medium and a scratch wound was made in each well by using a sterile 10 µl pipet-tip (Corning). At 1 h after wound induction, 10 µM bromodeoxyuridine (BrdU; Sigma) was added for 15 h to label mitotic cells. Cultures were subsequently washed with PBS and fixed with 4% PFA for 20 min, permeabilized with 0.5% Triton X-100 (Sigma-Aldrich) in PBS for 10 min, followed by 20 min incubation with 1 N HCl, and a 10 min neutralisation step with 0.1 M borate buffer and blocked with 3% BSA in PBS/0.5% Triton X-100 for 1 h. To identify BrdU, astrocytes were incubated with rat anti-BrdU (1∶100; Abcam) in PBS/0.5% Triton X-100 with 1% BSA 1 h at RT, washed with PBS/0.5% Triton X-100 followed by incubation with goat anti-rat FITC (1∶100: Jackson). Cells were washed twice with PBS/0.5% Triton X-100 once with PBS and embedded in Dapi-containing Vectashield (Vector Laboratories). Immunofluorescent staining was visualized using a Leica confocal microscope (Leica Microsystems). In five random 20× fields per culture condition, the number of proliferative cells (%) was calculated as follows: (number of BrdU positive cells/number of dapi positive nuclei)×100%.

### Visualization of F-actin and vinculin

The effect of inhibition of TG2 activity by KCC009 on focal adhesion formation was visualized by vinculin immunocytochemistry. Cells were plated on Fn-coated (2 µg/cm^2^, Sigma-Aldrich) 8-well chamber slides (Labtek, Nalge Nunc International, 2×10^4^ cells/well). After 24 h, medium was replaced by serum free medium and the cells were incubated at 37°C with cytokines for 48 h. Then, the cells were incubated with 0.5 mM KCC009 in 0.2% DMSO or 0.2% DMSO only (vehicle) in serum free medium for 1 h and subsequently fixed for 20 min with 4% PFA, permeabilized with 0.5% Triton X-100 (Sigma-Aldrich) in PBS for 10 min and blocked with 3% BSA in PBS/0.5% Triton X-100 for 1 h. To identify focal adhesions, astrocytes were incubated with mouse anti-vinculin antiserum (1∶400; Abcam) in PBS/0.5% Triton X-100 containing 1% BSA o/n at 4°C, washed with PBS/0.5% Triton X-100 followed by incubation with donkey anti-mouse Alexafluor-488 (1∶1,000; Invitrogen). Slides were washed and to identify F-actin cytoskeleton filaments, astrocytes were subsequently stained with rhodamine-phalloidin (1∶300; Invitrogen) for 1 h at RT, washed with PBS and embedded in Vectashield (Vector Laboratories). Immunofluorescent staining was visualized using a Leica confocal microscope (Leica Microsystems). Three independent experiments were performed.

### Statistics

Where appropriate, data were analyzed by one-way or two-way ANOVA, followed by a t-test for independent measurements (Fisher's LSD test). The statistical evaluation was carried out by using the NCSS 2007 statistical program (NCSS, East Kaysville, Utah, USA). Error bars represent standard error of the mean (s.e.m.).

## Results

### TG2 expression in primary rat astrocytes

**Figure 1:**
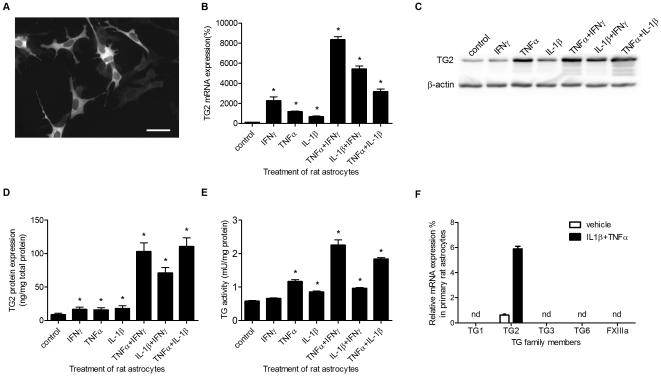
TG2 expression in primary rat astrocytes. **A**) TG2 is expressed in primary rat astrocytes as visualized by immunocytochemical analysis. Scale bar: 50 µm. **B**) TG2 mRNA expression after treatment with various single cytokines or with cytokine combinations. **C**) TG2 protein expression visualized by western-blot and **D**) measured with ELISA. **E**) TG2 activity levels measured with the TG Covtest TCMA. **F**) mRNA levels of transglutaminase (TG) family members in primary rat astrocytes. Data are expressed as mean+s.e.m., n = 6 per condition (F: n = 3 per condition), *P<0.05 compared to control, nd = not detectable.

TG2 immunoreactivity is present in primary rat astrocyte *in vitro*, but the intensity of the signal varied between cells (Fig. 1A). Since we were interested in the role of TG2 in astrocytes under inflammatory conditions, we studied the effect of various pro-inflammatory cytokines on TG2 levels and activity. TG2 mRNA levels were significantly increased after treatment with TNFα, IL-1β and IFNγ alone, while combinations of TNFα and IFNγ, or IFNγ and IL-1β showed a synergistic increase in TG2 mRNA transcript levels (Fig. 1B). TG2 protein levels could be visualised on western blot (Fig. 1C) and showed a clear increase after cytokine treatment, particularly after treatment with combinations of cytokines. TG2 protein levels were quantified using a specific TG2 ELISA. Like the mRNA levels, TG2 protein levels in astrocytes were significantly elevated after all single cytokine treatments (Fig. 1D). Moreover, all combined cytokine treatments induced a synergistic increased level of TG2 protein (Fig. 1D). Additionally, TG activity, present in cell lysates of these astrocytes, is significantly increased after treatment with TNFα and IL-1β alone, but not after treatment with IFNγ. However, the combined cytokine treatments all enhanced TG activity with TNFα and IFNγ giving a synergistic increase in TG activity (Fig. 1E). As treatment with a combination of cytokines resulted in the highest increase in TG2 expression and activity, the combination of TNFα and IL-1β was used to determine whether other TG family members are expressed by astrocytes. Of all members studied, TG2 mRNA could be detected under control conditions, which was enhanced after cytokine treatment (Fig. 1F) as shown earlier (Fig. 1B). Expression levels of TG1, TG3, TG6 and FXIIIa were undetectable, even after treatment of the cells with the combination of TNFα and IL-1β (Fig. 1F).

### Active TG2 on the surface of astrocytes

**Figure 2:**
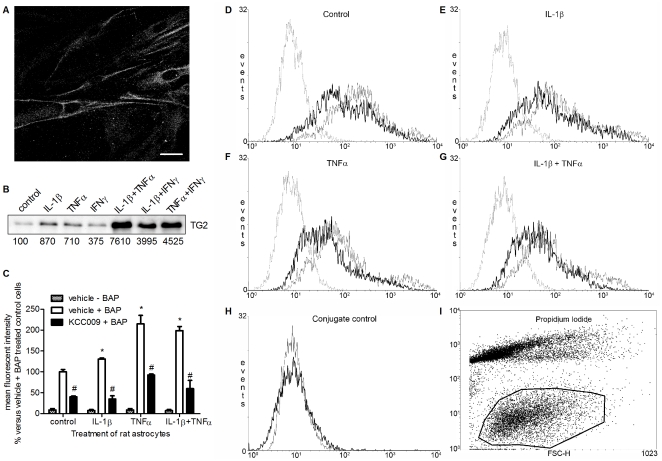
TG2 is expressed on the surface of rat astrocytes. **A**) Life non-permeabilized cells were immunofluorescently labeled to detect TG2 on the cell surface of rat astrocytes. Scale bar: 20 µm **B**) Cell surface-biotinylated proteins were immunoprecipitated, separated by SDS-PAGE, and immunoblotted for TG2. Bands were semi-quantified and expressed as % compared to control. **C**) FACS analysis of BAP incorporation on the surface of control rat astrocytes or astrocytes treated with IL-1β, TNFα or IL-1β+TNFα in the absence or presence of KCC009. Data represent mean fluorescent intensity from 3 independent experiments. *P<0.05 versus vehicle-treated control cells, ^#^P<0.05 versus vehicle-treated control or matched cytokine-treated. **D–G**) FACS plots of BAP incorporation on the surface of rat astrocytes **D**) untreated or treated with **E**) IL-1β, **F**) TNFα or **G**) IL-1β+TNFα and subsequent inhibition of TG2 activity. Dotted line: vehicle − BAP treated astrocytes; grey line: vehicle + BAP treated astrocytes; black line: KCC009 + BAP treated astrocytes **H**) Effect of conjugate (avidin-Alexa Fluor-488). Grey line: untreated cells –BAP−avidine-Alexa Fluor, black line: untreated cells –BAP+avidine-Alexa Fluor 488. **I**) Encircled astrocytes which were PI negative were gated for BAP.

Using immunocytochemistry on non-fixed cells, we were able to detect TG2 immunoreactivity located on or nearby the surface of primary rat astrocytes (Fig. 2A). To measure semi-quantitative changes in TG2 surface expression after treatment with cytokines, cell surface biotinylation followed by TG2 immunoprecipitation was performed. This resulted in a clear increase in the amount of TG2 protein present on the surface of astrocytes, again most dramatically when cells were treated with combinations of cytokines (Fig. 2B). Treatment with single cytokines resulted in an increased TG2 surface expression ranging from ∼4× to ∼9× the expression in untreated (control) astrocytes whereas treatment with the various combinations of cytokines resulted in a ∼40× to ∼76× elevated expression compared to untreated (control) cells. A treatment of single and combinations of IL-1β and TNFα were used for subsequent experiments since this combination was equally effective as the combination of TNFα and IFNγ in enhancing total TG2 protein level and single treatments with TNFα and IL-1β showed the highest increase in expression of surface TG2 which we considered of importance for interaction with Fn.

To determine whether this surface associated TG2 is active, we analyzed the incorporation of the competitive amine substrate BAP onto the surface of astrocytes using FACS analysis (Fig. 2C–G). Only cells negative for propidium iodide (PI) staining were gated for analysis of fluorescent intensity (encircled cells in Fig. 2I). The incorporation of BAP as measured by mean fluorescent intensity is depicted in Fig. 2C. Astrocytes incubated in the absence of BAP showed a background signal which is independent from the cytokine treatment (Fig. 2C, dotted line in Fig. 2D–G). BAP incorporation was already detectable in untreated (control) astrocytes and clearly increased with 30% or 115% after treatment with IL-1β or TNFα, respectively. Incubation of the astrocytes with the combination of IL-1β and TNFα resulted in a two-fold increase (200%) in mean fluorescent intensity (Fig. 2C, grey line in Fig. 2D–G). The incorporation of BAP was significantly reduced by 57% to 70% after treatment of the cells with the specific irreversible TG2 inhibitor KCC009 compared to vehicle-treated control or cytokine-treated astrocytes (Fig. 2C; black line in Fig. 2D–G). Addition of the conjugate avidin-Alexa Fluor 488 only to untreated astrocytes did not alter the fluorescent intensity of the cells compared to untreated cells incubated without avidin-Alexa Fluor 488 (Fig. 2H).

### Astrocyte-derived TG2 interacts with fibronectin

**Figure 3:**
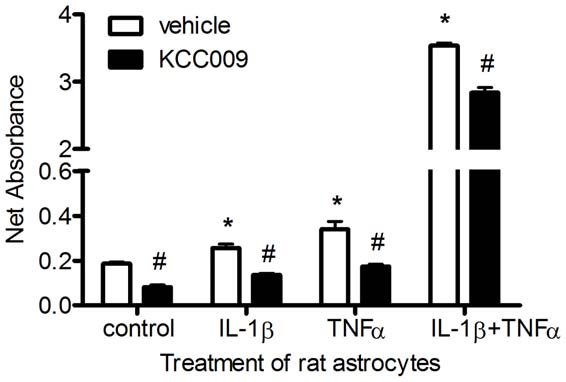
Incorporation of BAP into Fn by rat astrocyte-derived TG2. Data represent mean values ± s.e.m. from a representative experiment, n = 5 for each data point. *P<0.05 versus vehicle-treated control cells, ^#^P<0.05 versus vehicle-treated control or matched cytokine-treated cells.

Since TG2 was present and active on the surface of astrocytes, we questioned whether it plays a role in the interaction of the astrocytes with Fn. Therefore, we studied the incorporation of the competitive amine substrate BAP into Fn [28]. Astrocytes were treated with cytokines for 48 hours and subsequently, cells were plated onto Fn coated wells in serum free medium containing the competitive amine substrate BAP. After incubation, cells were extracted with deoxycholate containing buffer to eliminate the astrocytes leaving the Fn coating intact. BAP covalently incorporated by astrocyte-derived TG2 into extracellular Fn was quantified. Compared to vehicle-treated (control) cells, the amount of BAP incorporated into Fn was significantly increased by 1.5 or 2 times after treatment with TNFα or IL-1β, respectively (Fig. 3). The combination of these cytokines synergistically increased the amount of incorporated BAP by approximately 17 times (Fig. 3). To confirm that TG2 (partly) contributes to the BAP incorporation into Fn, rat astrocytes were co-incubated with the TG2 activity inhibitor KCC009. Incubation of cells with this inhibitor resulted in significantly reduced incorporation of BAP into Fn compared to matched vehicle-treated astrocytes (Fig. 3).

### Inhibition of TG2 activity reduced astrocyte adhesion onto fibronectin

**Figure 4:**
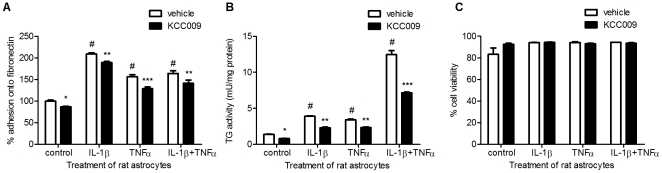
The effect of cytokine treatment and inhibition of TG2 activity on the adhesion capacity of astrocytes onto Fn. **A**) Adhesion of rat astrocytes onto Fn coated wells after treatment with cytokines and subsequent inhibition of TG2 activity. **B**) TG activity after treatment with cytokines and subsequent inhibition of TG2 activity. **C**) Cell viability measured using the propidium iodide assay. Data are expressed as mean+s.e.m., n = 15 (A) or n = 5 per condition (B,C), ^#^P<0.001 versus vehicle-treated control cells and *P<0.05, **P<0.01, ***P<0.001 versus vehicle-treated control or matched cytokine-treated cells.

To determine whether TG2 plays a role in adhesion of rat astrocytes onto Fn, a quantitative adhesion assay was performed. When astrocytes were cultured in the presence of IL-1β, TNFα or the combination of these cytokines, astrocyte adhesion onto Fn was increased by 110%, 56% or 64%, respectively compared to vehicle-treated (control) cells (Fig. 4A). Treatment with the TG2 activity inhibitor KCC009 significantly reduced the amount of adherent cells compared to vehicle-treated cells (Fig. 4A).

As a control, we checked whether KCC009 indeed reduced TG activity in astrocytes. We already showed in Fig. 2C that KCC009 treatment reduced TG2 activity on the surface of astrocytes. Indeed, co-incubation of the cytokines with KCC009 significantly reduced TG activity in the astrocytes, although it did not return to control levels (Fig. 4B). Of importance was that cytokine and/or KCC009 treatment did not affect astrocyte viability as measured with a propidium iodide assay (Fig. 4C).

### Inhibition of TG2 activity reduced migration of astrocytes across fibronectin

**Figure 5:**
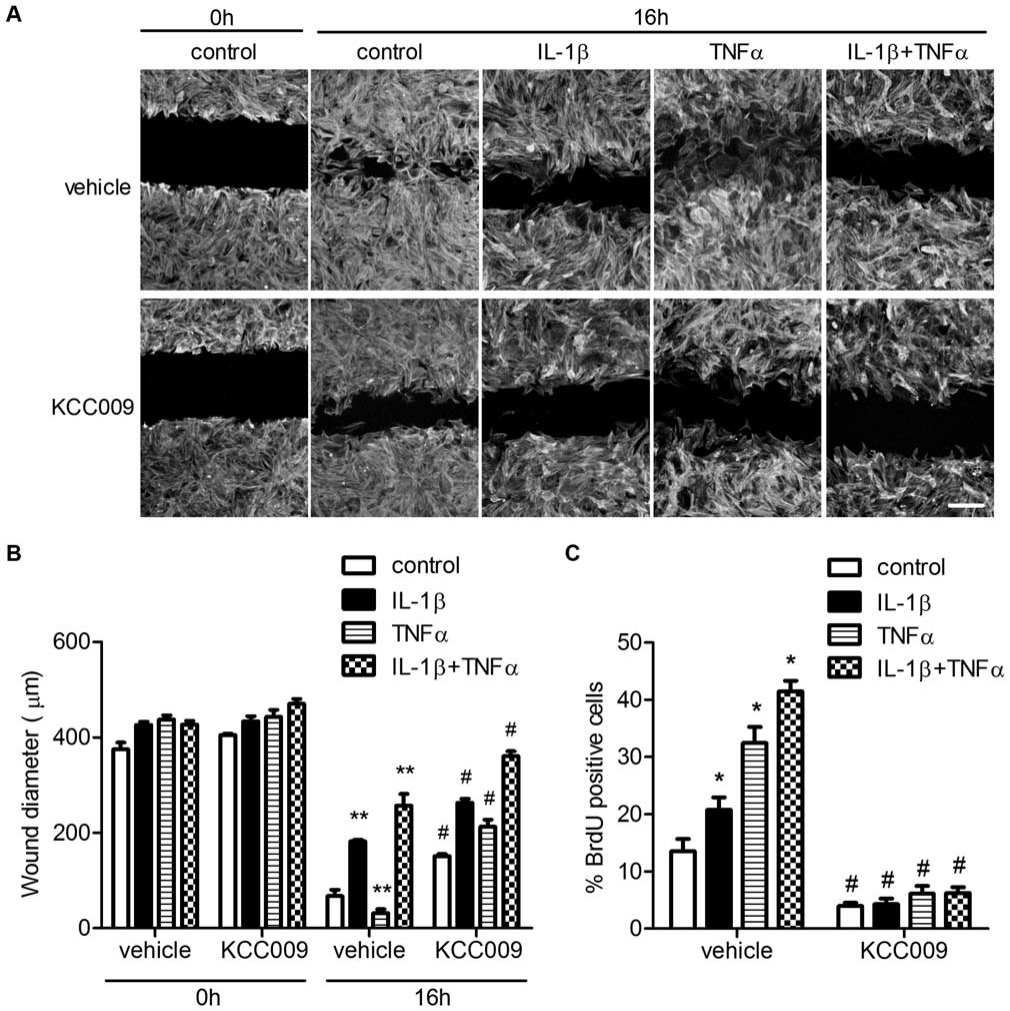
The effect of cytokine treatment and inhibition of TG2 activity on the migration capacity of astrocytes. **A**) A scratch wound was made in an astrocyte confluent cell layer plated onto Fn and astrocytes were allowed to migrate in the presence of vehicle or 0.5 mM KCC009. Cells were fixed after 0 and 16 h and stained with rhodamine-phalloidin to visualize the cells and wound diameter. Scale bar: 200 µm. **B**) Quantification of wound diameter after migration of rat astrocytes. Astrocytes were allowed to migrate for 16 h and the surface of the wound was quantified using phase-contrast microscopy after 0 and 16 h. **C**) BrdU incorporation after cytokine treatment and subsequent TG2 inhibition. Data represent mean+s.e.m. from a single experiment using 5 measurements per condition/time point and is representative out of 3 separate experiments. *P<0.05 versus vehicle-treated control cells, **P<0.01 versus vehicle-treated control cells, ^#^P<0.01 versus vehicle-treated control or matched cytokine-treated cells.

To analyze the involvement of TG2 in migration of cytokine-stimulated astrocytes, a quantitative migration assay was performed. Astrocytes were plated onto Fn coated wells and, after adherence, a scratch wound was induced to stimulate astrocyte migration in a monolayer. At first the migration of vehicle-treated control astrocytes was compared to vehicle-treated, cytokine-treated cells (Fig. 5A).

The initial wound diameter was similar for all conditions tested at t = 0 (∼400 µm). At 16 h after wounding, the diameter reduced to approximately 70 µm in vehicle-treated control astrocytes (Fig. 5B). Treatment with IL-1β, TNFα or the combination of IL-1β and TNFα resulted in an altered migration capacity of astrocytes (Fig. 5B). Cells cultured in the presence of TNFα showed an increased migration and reduced the gap to approximately 30 µm, whereas treatment with IL-1β or the combination of TNFα and IL-1β reduced astrocyte migration leaving a wound diameter of 180 µm and 260 µm respectively (Fig. 5B) Secondly, the migration of KCC009-treated astrocytes was compared to that of vehicle-treated cells. At 16 h after wounding, inhibition of TG2 activity with KCC009 in both control and cytokine-treated astrocytes significantly reduced migration compared to vehicle-treated control and matched cytokine-treated cells (Fig. 5A,B). Upon inducing the scratch, the Fn coating in the wells are damaged (Fig. S1). However, both the vehicle and KCC009-treated astrocytes are able to produce Fn and recover the damaged Fn layer (Fig. S1).

To determine whether altered migration was due to effects on cell proliferation, the nuclear uptake of BrdU was measured. Culturing the cells in the presence of IL-1β, TNFα or the combination of IL-1β and TNFα significantly increased the nuclear uptake of BrdU by 50%, 140% and 207%, respectively compared to vehicle-treated control astrocytes (Fig. 5C). Moreover, inhibition of TG2 activity by KCC009 significantly decreased BrdU uptake compared to vehicle-treated control and matched cytokine-treated cells (Fig. 5C). It is of interest to note that a similar reduced level of astrocyte proliferation was found independent of cytokine treatment.

### Cytokine induced TG2 activity is involved in focal adhesion formation

**Figure 6:**
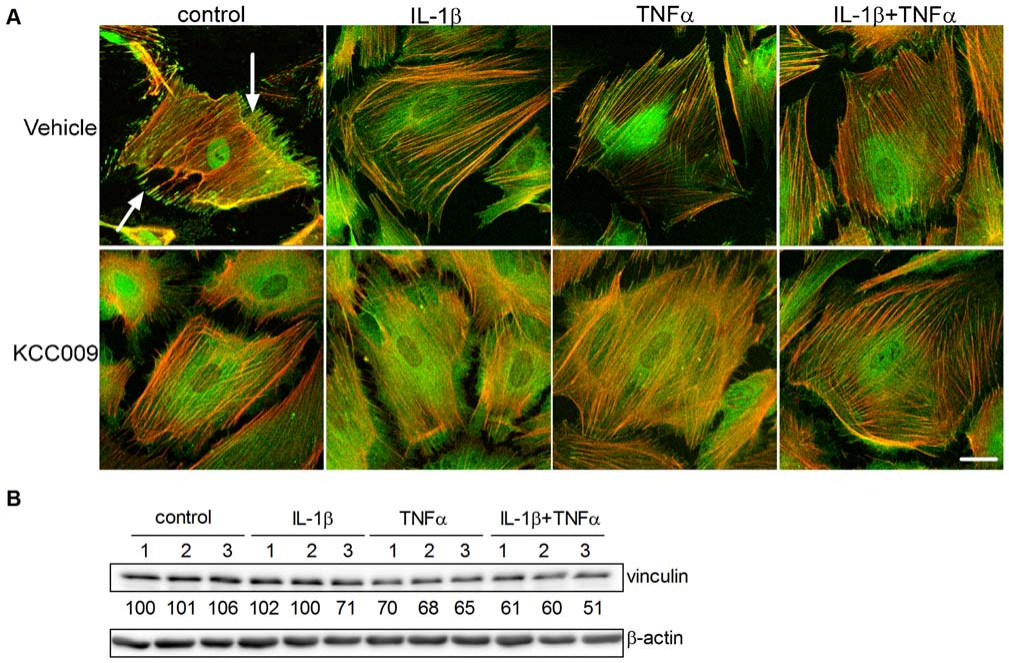
The effect of cytokine treatment and inhibition of TG2 activity on cytoskeletal rearrangements and RhoA activity. **A**) Confocal images of cells stained with rhodamin phalloidin (red) and an antibody against vinculin (green). Scale bar: 10 µm. **B**) Representative western blot of vinculin and β-Actin (n = 3). Semi-quantitative analysis of vinculin levels were corrected for β-Actin and the untreated control was set at 100%. The corrected levels are shown under the vinculin-blot. Cells were cultured in medium alone or medium containing IL-1β, TNFα or the combination for 48 h. Subsequently, cells were treated as follows: 1 = untreated, 2 = vehicle-treated, 3 = KCC009-treated.

Cellular adhesion and migration is partly mediated by an intracellular focal adhesion complex that links cell surface integrins to the actin cytoskeleton via integrin receptors and adaptor proteins such as vinculin [31]. F-actin was visualized using rhodamine-phalloidin and was organized in fine bundles that were orientated along the cellular axis. Vehicle-treated control astrocytes exhibit vinculin positive focal adhesions at the tip of F-actin stress fibers (Fig. 6A, arrows). When cells were cultured in the presence of IL-1β, TNFα or the combination of both cytokines, vinculin positive focal adhesions were still present, but less prominent, particularly after TNFα treatment (Fig. 6A). Compared to vehicle-treated cells, inhibition of TG2 activity with KCC009 induced a dramatic redistribution of immunoreactive vinculin, from a focal to a diffuse pattern (Fig. 6A) in both vehicle-treated and cytokine-treated astrocytes. Western blot analysis was performed to study the relative protein levels of vinculin. Bands were semi-quantified and corrected for β-actin expression levels. Treatment with IL-1β did not alter the level of vinculin protein. In contrast, vinculin protein levels were reduced after treatment with TNFα or the combination of IL-1β and TNFα (Fig. 6A,B). Treatment with KCC009 did neither affect vinculin levels in vehicle-treated nor in TNFα-treated astrocytes. KCC009 treatment only slightly reduced levels of vinculin protein in IL-1β and the combination of IL-1β and TNFα treated cells (Fig. 6B).

## Discussion

In the present study, we demonstrate that TG2 expression and activity is present in primary rat astrocytes and is increased after treatment with pro-inflammatory cytokines. Moreover, active TG2 is present on the surface of astrocytes, enhanced by cytokine exposure, and interacts with Fn to contribute to astrocyte adhesion and migration.

Our study provides evidence that pro-inflammatory cytokines, in particular TNFα, IL-1β and IFNγ or combinations of these cytokines enhance intracellular and cell-surface expression of TG2, even after two days of cytokine treatment. A relatively long-term expression of TG2 in these cells is of interest as under chronic neuropathological conditions TG2 appeared in astrocytes localized within and at the border of active MS lesions [29] and TG2 has been found in astrocytes surrounding lesions in experimental NeuroAIDS [32]. In active MS lesions, particularly leukocytes and lymphocytes produce inflammatory mediators, including IL-1β, TNFα and IFNγ. We therefore studied the effect of these cytokines on the expression and activity level of astrocytic TG2. As shown, these cytokines individually, and particularly combinations of them, enhance TG2 mRNA and protein levels as well as activity. So far, it has been reported that TNFα and particularly IL-1β elevate TG2 mRNA and protein levels in astrocytes [33]. The synergistically elevated levels of TG2 induced by treatment with combinations of cytokines are in concordance with observations on synergistic effects such as activation of the MAPK pathway and regulation of matrix metalloproteases and chemokines in various other cell types [34]–[36]. The molecular mechanisms underlying this synergistic effect of cytokines involve cooperative activation of transcription factors [37].

As we hypothesized that astrocyte-derived TG2 is involved in the interaction with the extracellular matrix, it is of importance to know whether TG2 is present on the cell surface. Indeed, TG2 protein is present on the astrocyte surface, and these TG2 surface levels are enhanced after exposure to the inflammatory cytokines. Previously, surface TG2 on epithelial cells was found to be increased by the cytokine TGFβ [38]. In addition, TG2 localized on the astrocyte surface is enzymatically active as determined by incorporation of BAP, an amine substrate for transglutaminases, using FACS analysis. TG activity on the astrocyte surface is also enhanced by treatment of the cells with inflammatory cytokines. To our knowledge, this is the first observation of TG activity on the astrocyte surface to be regulated by inflammatory cytokines. The fact that the observed mean fluorescence intensity could be greatly reduced by the TG2 specific inhibitor KCC009, indicates that TG activity on the surface of astrocytes is largely due to TG2. The observation that the fluorescence intensity of astrocytes incubated with the streptavidin-Alexa Fluor-488 conjugate is similar to that of completely untreated astrocytes indicates that with flow cytometry we solely detect BAP incorporation on the cell surface and not BAP incorporation inside the cell. This assay has also been used to study the cell surface levels of TG2 on the surface of other cells such as osteoblasts [39]. To determine whether this astrocyte-derived TG2 could play a role in ECM rearrangement, we studied the interaction between rat astrocytes and Fn, an important ECM protein in MS lesions [40]–[42]. We observed that the enhanced TG2 activity in and on the cell surface upon cytokine treatment was accompanied by an increased astrocyte adhesion onto Fn and that inhibition of TG2 activity, at least partly, reduced astrocyte adhesion. How astrocyte-derived TG2 contributes to the adhesion between astrocytes and Fn is uncertain. It could be due to high-affinity binding of surface TG2 to Fn [43] via interaction with its gelatin-binding domain [44], [45]. The interaction between astrocytes and Fn might also be attributed to cell surface TG2 complexed with β-integrins to enhance the affinity for binding to Fn as it has been shown for monocytes [44] and breast cancer cells [46]. Recently, it has been shown that the interaction of TG2 and Fn on the surface of osteoblasts was mediated via other surface receptors such as cell surface heparan sulphate proteoglycans including syndecan-2 and syndecan-4 [47]. Alternatively, TG2 present on the surface of endothelial cells has been demonstrated to contribute, via its cross-linking activity, to the interaction with Fn [28]. The observation that astrocyte-derived TG2 is able to incorporate BAP into Fn suggests that the active site of TG2, known to be involved in cross-linking activity, is of importance in the interaction between rat astrocytes and Fn. Still, we cannot exclude a contribution of direct binding between TG2 and Fn to play a role in the interaction between astrocytes and Fn.

As it is known that TG2 catalysed protein cross-links are stable to proteolytic and mechanical damage [21], TG2 activity on the surface of astrocytes might facilitate increased stability and resistance to degradation of modified ECM proteins such as Fn.

Rat astrocytes treated with IL-1β or the combination of IL-1β and TNFα displayed reduced migration across Fn which is in line with previous observations showing that treatment of human astrocytes with IL-1β attenuated migration across Fn [9]. In contrast, treatment with TNFα only resulted in an increased migration capacity of rat astrocytes as observed in other cell types, including cancer cells and mesenchymal stem cells [48], [49]. Taken together, our data suggest cytokine-specific regulation of astrocyte migration. This is confirmed by observations in neural precursor cells showing that IL-1β, but not TNFα, reduce migration of these cells [50].

The role of TG2 in astrocyte migration was determined by inhibiting its activity using KCC009. Reduction of TG2 activity in cytokine-treated rat astrocytes further decreased astrocyte migration capacity compared to matched vehicle-treated cells, suggesting that TG2 activity plays a role cell migration. It is good to note that if TG2 activity is indeed of importance in cell migration, then the observation that astrocytes treated either with IL-1β only or the combination of IL-1β and TNFα showed reduced migration is counterintuitive, because under these conditions TG2 activity is increased in and on the cells. Thus, our data indicate that TG2 is of importance in TNFα-induced migration of astrocytes, whereas its role in IL-1β-mediated effects on migration is probably less prominent.

The effect of cytokines on astrocyte migration is unlikely to be attributed to altered proliferation, because the combination of IL-1β and TNFβ showed the least migration, but most proliferation of astrocytes. Moreover, inhibition of TG2 activity by KCC009 similarly attenuated astrocyte proliferation independent of cytokine treatment, which is thus not responsible for the cytokine-specific effects on migration observed. Of importance to note is that upon a scratch wound is made, the Fn matrix is damaged (Fig. S1, vehicle t = 0 h). Upon longer incubation, the primary rat astrocytes were able to produce and deposit Fn to a similar extent after treatment with vehicle or KCC009 (Fig. S1, vehicle or KCC009 t = 24 h, left side of dotted lines). However, KCC009-treated astrocytes migrate less, and thus deposit less Fn (Fig. S1, vehicle or KCC009 t = 24 h, right side of dotted lines, arrows). We thus consider that KCC009 does not clearly affect the assembly of Fn, but rather affects TG2 activity in and on the astrocytes which is of importance for cell migration.

Astrocyte adhesion and migration are multistep processes involving formation and stabilisation of focal adhesions which are necessary for cell adhesion, and dissolution of focal adhesions to mediate cell migration. These focal adhesions link the extracellular matrix to the cytoskeleton via integrin receptors and adapter proteins such as vinculin [31]. Treatment with TNFα or the combination of IL-1β and TNFα reduced vinculin protein levels, whereas treatment with IL-1β had no effect. This may explain our observation that IL-1β-treated astrocytes show little migration, whereas loss of vinculin, indicative for dissolution of focal adhesions is observed in TNFα-treated astrocytes that do migrate. Immunocytochemically, vinculin immunoreactivity seemed, although not quantified, less prominent at the tip of F-actin fibers of TNFα-treated astrocytes, particularly. This again supports the positive effect of TNFα on astrocyte migration. Our data are in agreement with the observation that TNFα regulates cytoskeletal organization and dispersion of vinculin from focal adhesion sites, resulting in increased migration of smooth muscle cells [51].

After inhibition of TG2 activity by KCC009 treatment, vinculin protein levels were hardly affected, suggesting that cytokine-enhanced TG2 does not clearly contribute to the reduction in vinculin protein levels. However, inhibition of TG2 activity by KCC009 does result in a dramatic intracellular redistribution of vinculin as observed immunocytochemically, from a focal to a diffuse cytoplasmic localization pattern. Now under all conditions, the localization of vinculin at the tip of F-actin fibers has disappeared. Unexpectedly, migration of astrocytes is not enhanced under these conditions. The reduced TG2 activity resulting in the intracellular redistribution of vinculin may now have affected the focal adhesions in such a way that migration is impaired. Thus, TG2 is an important factor in necessary for interaction of astrocytes with the ECM, i.e. Fn.

All together, our data support a role for TG2 in astrocyte adhesion and migration, but we cannot conclude whether surface-associated TG2 or intracellular TG2 plays a more prominent role in these processes. It has been shown that intracellular TG2 can induce activation of NFκB, an inflammatory transcription factor, which in turn can regulate downstream processes such as cell survival and spreading [52], [53]. Moreover, enhanced nitric oxide levels in fibroblasts reduced intracellular and extracellular TG2 activity and subsequent activation of TGFβ in an NFκB dependent manner, ultimately resulting in reduced ECM remodelling [19]. Furthermore, activation of focal adhesion kinase (FAK) has been linked to TG2 expression that results in increased motility, independent of surface expression of TG2 in tumor cells [54]. However, in osteoblasts and mesenchymal stem cells it was shown that FAK activation and subsequent effects on adhesion and migration is dependent on cell surface TG2 activity levels [47], [55].

In summary, we have shown that TG2 expression and activity present in and on the surface of rat astrocytes is enhanced upon cytokine treatment. This coincides with an increased adhesion to and interaction of the astrocytes with the ECM protein Fn. Inhibition of TG2 activity reduced the adhesion of astrocytes onto Fn. Interestingly, cytokine-specific effects on cell migration were observed, but inhibition of TG2 activity reduced astrocyte migration independent of cytokine treatment. Furthermore, inhibition of TG2 activity resulted in altered focal adhesion formation which is necessary for astrocyte-ECM interaction. All together, we put forward that under chronic neuroinflammatory conditions, e.g. MS, TG2 activity in and on the surface of astrocytes is involved in remodeling of the ECM, in particular by interacting with Fn and possibly other ECM proteins. Concomittant effects on astrocyte adhesion and migration may ultimately facilitate glial scar formation.

## Supporting Information

Figure S1 **Presence of fibronectin besides and in the scratched wound before and after migration of primary rat astrocytes.** Primary rat astrocytes were plated onto fibronectin coated wells and treated with cytokines (IL-1β+TNFα, 50 ng/ml each) for 48 hours. Then, a scratch wound was made and cells were allowed to migrate in the presence of vehicle or 0.5 mM KCC009. At 0 and 24 h after wound induction, the astrocytes were eliminated using 0.1% sodium-deoxycholate (Sigma) for 20 min. The “empty” wells were subsequently fixed with 4% paraformaldehyde, and stained for fibronectin (rabbit anti-human fibronectin, 1∶1,000; Sigma-Aldrich). The presence of fibronectin besides (at the left side of the dotted line) and in the wound (at the right side of the dotted line) was visualized. Fibronectin was reduced at the site of the wound after inducing a scratch wound (t = 0) but produced and deposited again by vehicle and KCC009 treated astrocytes (t = 24 h, vehicle and KCC009, left from dotted line). However, the KCC009-treated astrocytes migrated to a lesser extent than vehicle-treated astrocytes, and thus less Fn is present (t = 24 h, vehicle and KCC009, right from dotted line). Scale bar: 200 µm.
(TIF)
