# Supplementary figures and images for: Astrocyte-Derived Tissue Transglutaminase Interacts with Fibronectin: A Role in Astrocyte Adhesion and Migration?
Source: PLoS One. 2011 Sep 16;6(9):e25037. doi: 10.1371/journal.pone.0025037 (PMC3174992; doi:10.1371/journal.pone.0025037)

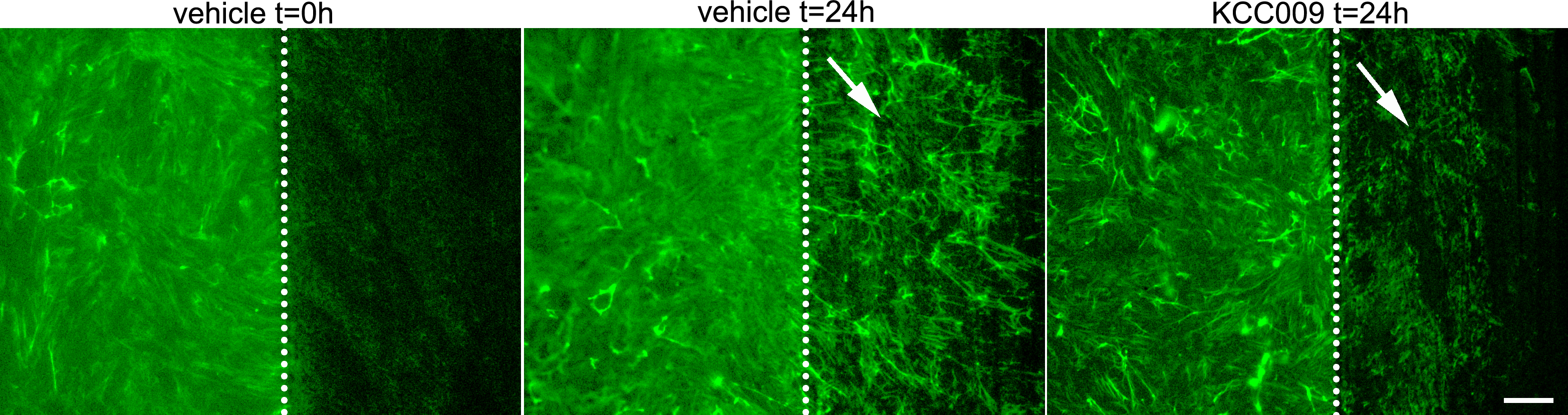

Supplement: Figure S1 — Presence of fibronectin besides and in the scratched wound before and after migration of primary rat astrocytes. Primary rat astrocytes were plated onto fibronectin coated wells and treated with cytokines (IL-1β+TNFα, 50 ng/ml each) for 48 hours. Then, a scratch wound was made and cells were allowed to migrate in the presence of vehicle or 0.5 mM KCC009. At 0 and 24 h after wound induction, the astrocytes were eliminated using 0.1% sodium-deoxycholate (Sigma) for 20 min. The “empty” wells were subsequently fixed with 4% paraformaldehyde, and stained for fibronectin (rabbit anti-human fibronectin, 1∶1,000; Sigma-Aldrich). The presence of fibronectin besides (at the left side of the dotted line) and in the wound (at the right side of the dotted line) was visualized. Fibronectin was reduced at the site of the wound after inducing a scratch wound (t = 0) but produced and deposited again by vehicle and KCC009 treated astrocytes (t = 24 h, vehicle and KCC009, left from dotted line). However, the KCC009-treated astrocytes migrated to a lesser extent than vehicle-treated astrocytes, and thus less Fn is present (t = 24 h, vehicle and KCC009, right from dotted line). Scale bar: 200 µm. (TIF) [file pone.0025037.s001.tif]
